# Use of spinal anaesthesia in neonates and infants in Antananarivo, Madagascar: a retrospective descriptive study

**DOI:** 10.1186/s13104-020-05330-9

**Published:** 2020-10-21

**Authors:** Harifetra M. R. Randriamizao, Aurélia Rakotondrainibe, Lova D. E. Razafindrabekoto, Prisca F. Ravoaviarivelo, Andriambelo T. Rajaonera, Mamy L. Andriamanarivo

**Affiliations:** 1 Department of Anesthesia and Intensive Care, University of Antananarivo, Antananarivo, Madagascar; 2 Department of Anesthesia and Intensive Care, University of Fianarantsoa, Fianarantsoa, Madagascar; 3 Department of Anesthesia and Intensive Care – Operating Theater, CHU Joseph Ravoahangy Andrianavalona, Antananarivo, Madagascar; 4 Department of Pediatric Surgery, University of Antananarivo, Antananarivo, Madagascar; 5 Surgical Intensive Care, CHU Joseph Ravoahangy Andrianavalona, BP 4150 – 101, Antananarivo, Madagascar

**Keywords:** Spinal anesthesia, Newborns, Infants, Preterm, Ex prematures, Madagascar

## Abstract

**Objective:**

The aim of this study was to present the first cases of spinal anesthesia, in newborns and infants, preterm/ex-prematures, in order to determine its feasibility and its potential harmlessness, in Antananarivo—Madagascar. Indeed, spinal anesthesia is a low cost technique and can limit respiratory complications, postoperative apnea *a contrario* with pediatric general anesthesia which can lead to perioperative risks.

**Results:**

In a retrospective, descriptive, 7-year (2013 to 2019) period study, conducted in the University Hospital Joseph Ravoahangy Andrianavalona, 69 patients’ data files planned to have spinal anesthesia were recorded. These pediatric patients were predominantly male (sex ratio = 2.8) and 37 [28–52] days old. The smallest anesthetized child weighed 880 g; the youngest was 4 days old. Twenty-seven (27) of them were premature and 20.3% presented respiratory diseases. They were mostly scheduled for hernia repair (90%). Spinal anesthesia was performed, with a Gauge 25 Quincke spinal needle, after 2 [1–2] attempts with hyperbaric bupivacaine of 4 [3.5–4] mg. Failure rate was 5.8%. The heart rate was stable throughout perioperative period and no complications were observed.

## Introduction

Spinal anesthesia (SA) is a part of anesthesia for sub umbilical and lower limb surgeries [1]. The first spinal anesthesia in children had been practiced by Bier in the nineteenth century (1898), then by Bainbridge (1901) and Gray (1909) [1, 2]. Due to considerable improvements of general anesthesia (GA) in the middle of twentieth century, this regional anesthesia was abandoned [2]. In 1990–2000, spinal anesthesia in newborns or in preterm had an upsurge of 2.1 to 3.6% in regional anesthesia after the decline of caudal anesthesia practice [3, 4]. Nowadays, SA tends to be mainly performed in pediatric anesthesia, up to 95.4% of children, as much in the newborns as in the preterm [1–3, 5]. SA allows the prevention and the reduction of perioperative complications even if its duration is an important limiting factor [1–3, 6]. Because of this limitation, short surgery is the most indicated under SA [1, 5, 7]. For more efficiency, this technique should be performed by experimented anesthetists [1, 8].

In Antananarivo—Madagascar, at the Hospital University of JR Andrianavalona (CHU JRA), spinal anesthesia has been performed since 2013. The aim of this study is to present the first cases of spinal anesthesia, in newborns and infants, preterm / ex-prematures, in order to determine its feasibility and its potential harmlessness, in Antananarivo.

## Main text

### Methods

#### Study design

A retrospective, descriptive study was conducted on the data files from scheduled pediatric surgeries under spinal anesthesia. The study was conducted, in the operating theater of the CHU JRA, from 2013 to 2019. This latter is the surgical reference center of Madagascar, particularly in pediatric surgery.

This observational, retrospective study conducted in operating theater had the approval of the University Hospital Joseph Ravoahangy Andrianavalona and the Department of Anesthesia and Resuscitation of the Faculty of Medicine of Antananarivo.

During the perioperative procedures, the general anesthesia risks and modalities of spinal anesthesia were explained to the patients’ parents, during the anesthesia consultation (AC). Their written informed consent was obtained for the anesthesia and surgery and included in the anesthesia file. The AC was carried out and validated by anesthesiologists (also the performers of the SA) who had prior training about SA in small children.

#### Anesthesia procedures

The lumbar puncture was considered successful when the cerebrospinal fluid (CSF) flowed back. Then, the patient was placed in a 45° tilt-head up. The local anesthetic (LA) used was hyperbaric 0.5% bupivacaine (1 mg/kg = 0.2 mL/kg). Spinal anesthesia was considered successful, when the patient no longer moved his lower limbs or had anal sphincter relaxation; and also when GA conversion or complementary local anesthesia (by the surgeon) throughout the surgical procedure was not required. When spinal anesthesia succeeded, a pacifier dipper with sugar water was given to the baby.

#### Studied parameters and variables

The studied variables and parameters were (i) gender, (ii) perinatal parameters: weeks at birth, prematurity (with causes), birth weight, (iii) parameters during the AC: age (in days), postconceptual age (in corrected weeks [CW] for preterm or ex prematures), and weight, (iv) spinal anesthesia parameters: procedure of lumbar puncture and spinal anesthesia, (v) perioperative variables: complementary procedures during SA, surgery characteristics, length of stay in the postoperative recovery room and (vi) perioperative heart rate.

#### Presentation of data

The continuous variables are expressed in median [interquartile 25%–75%] and the categorical variables in frequencies.

### Results

Over the 7-years period, 69 SA were indicated (Additional file 1: Figure S1: Spinal anesthesia procedure—CHU JRA) in predominantly male (*sex ratio* = 2.8) and 37 [28–52] days old patients (Table 1). The smallest patient weighed 880 g and the youngest was 4 days old. The patients weighed 2400 [1995–3025] g at birth and 3450 [2800–4240] g on the AC day. Twenty-seven patients were preterm, aged 33 [27–37] weeks at birth and with a corrected age of 40.5 [37–42] CW on the anesthesia day. Fourteen children (20.3%) had a medical history of respiratory diseases. The intervention was performed 5 [3, 4] days after the AC. The main surgery indications (Table 2) were hernias and surgical procedure was 27.5 [17.5–40.0] minutes ranging from 10 to 65 min. For all patients, perioperative perfusion was performed with a G24 intravenous catheter. The lumbar puncture was performed on a curved back patient, in a sitting or lateral decubitus (if sedated) position, by a trained nurse anesthetist. The puncture was performed at the intersection point between the line connecting the highest point of both iliac crests and the vertebral axis. The localization of lumbar puncture was determined by palpation, by the SA performer. The material used was an 80 mm—G25 Quincke spinal needle (the thinnest needle available at the CHU JRA). Inhaled sedation was needed in 13.0%, when positioning patient was difficult. Number of punctures was 2 [1, 2] attempts. A dose of 4 [3.5–4] mg hyperbaric 0.5% was administered. The lumbar puncture was successful in 97.1% and spinal anesthesia in 94.2%. No complementary local anesthesia by the surgeon was required. The GA conversion was 5.8% when SA failed.

**Table 1:** Population study characteristics

		N	%
Pediatric patients	Newborns	18	26.0
	Infants	51	73.9
Preterm or ex-prematures		27	39.1
Weight at the anesthesia consultation (g)	< 1000	1	1.4
	[1000–2000[	2	2.9
	[2000–3000[	17	24.6
	[3000–4000[	24	34.8
	[4000–5000[	16	23.2
	[5000–6000[	7	10.1
	≥ 6000	2	1.4
Medical history	Respiratory diseases^a^	14	20.3
	Resuscitation at birth	3	4.3
	Incubator after birth	6	8.7
	Others^b^	3	4.3
Causes of prematurity	Anamnios or Oligoamnios	4	5.8
	Gestational diabetes and/or pregnancy-induced hypertension	8	11.6
	Pre-eclampsia or Eclampsia^c^	6	8.7
	Maternal fetal infections	4	5.8
	*Placenta prævia*	1	1.4
	Twin pregnancy	1	1.4
	PROM^d^ + cord prolapse	1	1.4
	Fetal anoxia^c^	2	2.9

^a^Respiratory diseases = meconial amniotic fluid inhalation at birth, bronchiolitis; ^b^Others = intrauterine growth restriction (IUGR), neonatal infection, gastroesophageal reflux; ^c^ ± other conditions (preeclampsia, twin pregnancy); ^d^PROM = premature rupture of fetal membranes

**Table 2:** Perioperative characteristics

		N	%
Surgery	Hernia (inguinal and/or scrotal) ± circumcision	50	72.5
	Bilateral hernia	*26*	*37.7*
	Right hernia	*16*	*23.2*
	Left hernia	*8*	*11.6*
	Bilateral ovarian hernia	19	27.5
	Surgery of lower limbs (gangrene/necrosis)	2	2.9
Spinal anesthesia (SA)	Position of the patient		
	Lateral decubitus	9	13.0
	Sitting position	60	87.0
	Number of punctures		
	1	34	47.3
	2	22	31.9
	≥ 3	13	18.8
	Incidents during the technique (blood reflux)		
	CSF reflux after a first blood reflux	1	1.4
	CSF reflux after a 2nd puncture	4	5.8
	Failure of SA		
	Due to the technique^a^	2	
	Due to the LA	2	2.9
	General anesthesia (GA) conversion	4	5.8 2.9

CSF: Cerebral Spinal Fluid; LA: Local Anesthetic; GA: General Anesthesia

^a^Blood reflux without secondary CSF reflux or after 2nd lumbar puncture

**Fig. 1:**
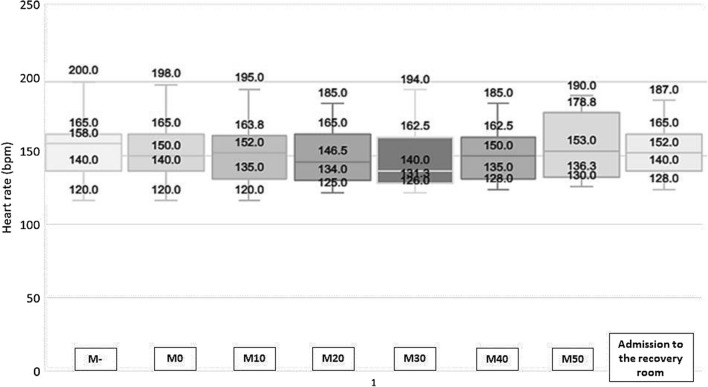
Perioperative heart rate of the patients under spinal anesthesia (expressed in median, interquartile 25%-75%, minimum and maximum). *bpm* = *beat per minute; M* = *minute; M-* = *heart rate before SA*

The heart rates were slight constant throughout the intervention until admission to the recovery room (Fig. 1). The patients stayed in postoperative recovery room during 70 [60–120] minutes. No perioperative complications were observed.

### Discussion

In the present study, spinal anesthesia was scheduled for 69 babies. This series represents the first pediatric spinal anesthesia, performed since 2013, in Madagascar (Additional file 2: Data and materials). In developed countries, such as in the United States, SA has been included since 1977, with 262 SA, on less than 1-year patients, in 15 years [9]. Williams et al. [5] reported 95.4% pediatric spinal anesthesia. In Europe, 400 to 500 SA are performed annually, (18% in preterm and 5% newborns) [3, 10]. In other countries, like India, in a 1-year period study, 102 children (from 6 months to 14 years) received spinal anesthesia for sub umbilical and lower limb orthopedic surgeries [6]. In low-income countries, few studies on pediatric spinal anesthesia have been related. Ela A.A. et al*.* [11], in Cameroon, report a series of 55 children operated under spinal anesthesia. However, the use of spinal anesthesia especially in “precarious” or “difficult” situations is attractive because it requires fewer perioperative resources [12].

Spinal anesthesia in the present study was performed, even at very young age (27 preterm) and low weight patients, and 14 children had a medical history of respiratory diseases. Spinal anesthesia is primarily indicated when general anesthesia presents a high risk (= respiratory complications or postoperative apnea because of pulmonary disease or prematurity) [1, 7, 10, 13]. SA is the “gold standard” technique in preterm (gestational age ≤ 37 weeks) and high-risk patients (preterm infants with postconceptual age < 60 CW) [2, 9]. Indeed, this population is at high risk of postoperative apnea, especially if general anesthesia is performed. Spinal anesthesia is a safe alternative when tracheal intubation should be avoided (due to bronchopulmonary dysplasia or respiratory diseases …) [1, 4]. Indeed, spinal anesthesia can reduce or avoid apnea [9, 10]. Also, SA causes minimum respiratory complications [2, 10, 14, 15]. In this study, most of the patients had respiratory diseases (20.3% rhino-bronchitis) and 39.1% were premature. In the present study, all these facts motivated spinal anesthesia. In addition, patients’ characteristics were quite similar to a study by Hermanns et al. [13]: 34.5 (24–40) weeks at birth, 10 (5–24) weeks postnatal age at the time of the intervention, and 3.5 (2.2–5.2) kg in weight.

The surgeries (lasting 27.5 [17.5–40.0] min), in the present study, were mostly hernia repairs. Spinal anesthesia is the gold standard for lower abdominal and lower limbs surgeries under 90 min duration [1, 2, 5, 7]. This was similar to a study of Ela et al. [11] (from 25 to 78 min) and shorter than results in a study of Frumiento et al*.* [9] (48 [15–130] min). The most concerned surgeries are inguinal hernia repair [1, 2, 5, 7]. But other surgeries (resection of ileostoma, sacral teratoma …) can also be performed under SA [11, 13].

The spinal puncture (2 [1, 2] attempts) was performed in sitting or lateral position, in the intersection point between the line connecting the highest point of both iliac crests (Tuffier’s line) and the vertebral axis, with an 80 mm—G25 Quincke spinal needle. This midline approach is the most used in SA in small children, in lateral or seated position [6, 13, 16]. A 25G pencil-point needle such Whitacre (avoiding post lumbar puncture headache) or 25G neonatal spinal needle are recommended [11, 13]. These types of needles are not available in the CHU JRA, so 25G Quincke spinal needle was used for all patients.

Hyperbaric bupivacaine 0.5% was used with a dose of 4 [3.5–4] mg. The most used local anesthetics are tetracaine 0.5% and bupivacaine 0.5% lasting 90 to 120 min [1]. Hyperbaric bupivacaine (0.5%) is mostly used in a dose from 0.3 to 1 mg/kg [1, 6, 13, 17].

In the present study, for all patients, 2 anesthetists who had prior training on this technique performed the SA to limit performance bias. Even the spinal anesthesia can be performed by either an anesthetist-intensivist, or an anesthesia-intensivist trainee, or a state-certified nurse anesthetist, SA performer should be well trained for the technique [11]. Trainees in anesthesia have a significant different success rate compared to anesthesiologists (83% versus 98.9%); the failure rate is 28% and the risk of total spinal anesthesia is approximately 0.63 to 0.8%, if the performer is not trained [1, 5].

The success of the lumbar puncture was 97.1% after 2 [1, 2] attempts and SA success was 94.2%. Since the Bromage score is not assessable among this pediatric population, the success of the spinal anesthesia is estimated and based on the sudden loss of leg movement while normal tonus in the arms and/or the relaxation of the anal sphincter and the possibility of performing the surgical procedure [14, 18]. Williams et al. [5] reported a success rate of 97.4%, quite similar with our results. In the present study, no complementary local anesthesia by the surgeon was required and the GA conversion was 5.8%. This failure rate was similar to literature, varying from 1.04 to 24.6% [7, 9, 17, 19]. Dohms et al. [19] find a failure rate of 7.5% and 16% required supplemental anesthesia, also more than two punctures were needed in 28%. In Kachko et al.'s [7] study, conversion to general anesthesia was 1.04%. In inguinal hernia repair, Frumiento et al. [9] describe 91.4% of adequate spinal anesthesia, 78.6% no supplemental anesthesia, 4.5% complementary local anesthesia and 2.2% general anesthesia conversion.

The heart rates were stable throughout the perioperative period. Spinal anesthesia allows remarkable cardiovascular stability and can avoid bradycardia with minimum cardiac complications [2, 8, 10, 14, 15, 18]. But in some cases, 1.5% patients experienced bradycardia in the operating room, and 1.9% received vagolytics [9].

Spinal anesthesia offers a good balance between safety and perioperative risks and appears to be a safe technique, provided that the contraindications are respected; the frequency of complications is 30% [1, 5, 10, 15]. SA causes less bradycardia, apnea, desaturation, requiring postoperative respiratory assistance than GA; ventilation and oxygenation are not generally compromised, even in patients at high risk [8, 18, 20]. In the present study, no perioperative complications were observed.

In Antananarivo, this series is the first to have been reported. The strength of this study is the characteristics of the population (preterm, newborns, and infants).

### Conclusion

Spinal anesthesia can be performed on small pediatric patients, even on very young and very low-weight patient. Hernia repair was the most concerned surgery under spinal anesthesia which had a high success rate and no consequent complications. These first cases should motivate a wider and more frequent practice in Antananarivo, Madagascar.

## Limitations

The monocentric and retrospective characteristic of this study are the main limits; the presented results do not reflect the whole Malagasy population.

## Supplementary information

**Additional file 1: Figure S1.** Spinal anesthesia procedure at the CHU JRA (**A**: Materials used = hyperbaric bupivacaine, 1 mL syringe, Q25G spinal needle;** B**: Sitting position;** C**: Lateral decubitus position;** D**: Lumbar puncture).

**Additional file 2.** Data and materials

## Data Availability

Data and materials are available (excel) Additional file 2.

## Abbreviations

AC: Anesthesia consultation
bpm: Heart beat per minute
CHU JRA: Centre Hospitalier Universitaire Joseph Ravoahangy Andrianavalona
CSF: Cerebrospinal fluid
CW: Corrected weeks
G: Gauge
GA: General anesthesia
IUGR: Intrauterine growth restriction
kg: Kilogram
LA: Local anesthetic
mg: Milligrams
mL: Milliliters
PROM: Premature rupture of fetal membranes
SA: Spinal anesthesia
