# Supplementary material for: Use of spinal anaesthesia in neonates and infants in Antananarivo, Madagascar: a retrospective descriptive study
Source: BMC Res Notes. 2020 Oct 21;13:491. doi: 10.1186/s13104-020-05330-9 (PMC7579963; doi:10.1186/s13104-020-05330-9)
Supplement: Supplementary file 1 — Additional file 1: Figure S1. Spinal anesthesia procedure at the CHU JRA (A: Materials used = hyperbaric bupivacaine, 1 mL syringe, Q25G spinal needle; B: Sitting position; C: Lateral decubitus position; D: Lumbar puncture). [file 13104_2020_5330_MOESM1_ESM.docx]

**FIGURE S1**


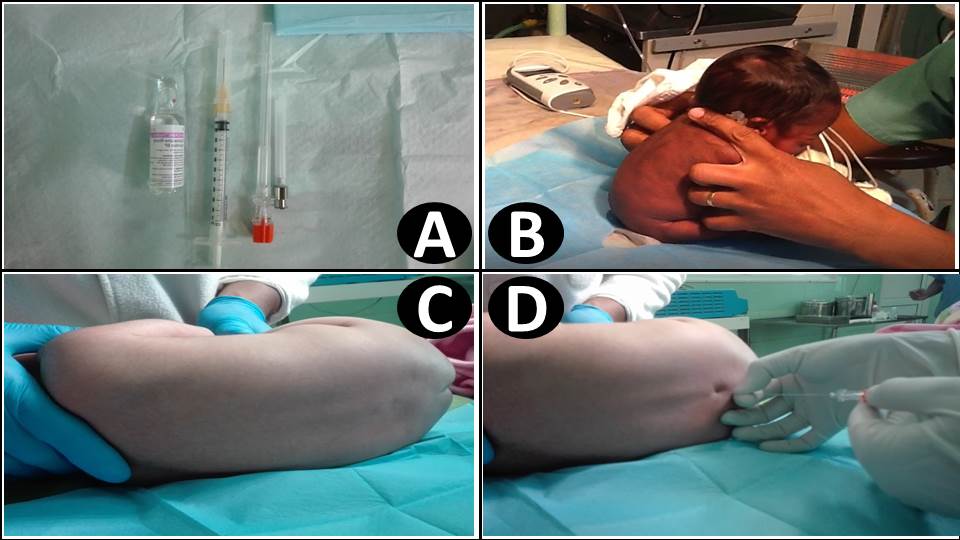


*Figure S1*: Spinal anesthesia procedure at the CHU JRA.

(**A**: Materials used = hyperbaric bupivacaine, 1 mL syringe, Q25G spinal needle; **B**: Sitting position; **C**: Lateral decubitus position; **D**: Lumbar puncture).
